# The Prognostic Index Independently Predicts Survival in Patients with Pancreatic Ductal Adenocarcinoma Undergoing Resection

**DOI:** 10.1245/s10434-019-08161-6

**Published:** 2020-01-03

**Authors:** Elisabeth S. Gruber, Gerd Jomrich, Alexandra Kaider, Michael Gnant, Klaus Sahora, Martin Schindl

**Affiliations:** 1 Pancreatic Cancer Unit, Division of General Surgery, Department of Surgery, Comprehensive Cancer Center, Medical University of Vienna, Vienna, Austria; 2 Section for Clinical Biometrics, Center for Medical Statistics, Informatics, and Intelligent Systems, Medical University of Vienna, Vienna, Austria

## Abstract

**Background:**

Cancer-related inflammation is associated with tumour proliferation, maintenance and dissemination. It therefore impacts pancreatic cancer survival. The goal of this study was to examine the Prognostic Index (PI) as a prognostic biomarker for survival in patients with pancreatic ductal adenocarcinoma (PDAC). In addition, we explored factors known to interact with the immune and inflammation cascade that might interfere with the PI’s strength for prognostication.

**Methods:**

Patients with PDAC undergoing resection were analysed retrospectively. The PI was calculated from preoperatively derived C-reactive protein levels and white blood count. Data were subject to correlation and survival analysis.

**Results:**

Of 357 patients, 235 (65.8%) patients had a PI 0, 108 (30.3%) PI 1, and 14 (3.9%) PI 2. Median (quartiles) survival with a high PI (group 1 + 2) was 13.2 months (7.7–27.0), compared with 18.7 months (10.2–35.4) with a low PI (group 0; *p *= 0.012). The PI proved to be an independent prognostic factor for cancer-specific survival (*p *= 0.003) adjusted for conventional prognostic factors. Prognostic strength was influenced by the presence of a bile stent (*p *= 0.032).

**Conclusions:**

The PI is a strong and solid independent prognostic tool for survival in patients with PDAC undergoing resection. Preoperative survey of inflammatory activity as provided by the use of a biomarker like the PI may help to identify those patients at risk of a poor prognosis.

Pancreatic ductal adenocarcinoma (PDAC) remains one of the solid malignancies with a poor prognosis despite some improvements gained over the course of centuries past. This is because the majority of patients were diagnosed with a locally advanced or metastatic disease.^1^ One key factor for adequate treatment is reliable, detailed knowledge of the actual extent of disease and the patient’s condition at initial diagnosis. To date, this was based mainly on the radiographic description of vascular involvement by the tumour and the presence of distant metastases. Recently, factors reflecting tumour biology, such as CA19-9 serum concentration and suspected lymphadenopathy as well as patients’ condition, were added to the clinical estimation of disease stage and outcome.^2^^–^5 However, the body’s inflammatory response to the presence of a malignancy was frequently neglected, despite that it showed a significant impact on outcome in PDAC.^6^^–^10

Among a wide range of inflammatory mediators, cytokines stimulate tumour development at different stages from tumourigenesis to dissemination. In addition, they affect treatment response and are associated with rapid deterioration and a dismal prognosis.^11^^–^14 In clinical practice, this is simply reflected by the production of acute phase proteins, such as C-reactive protein (CRP) and the increase of circulating blood leucocytes.^15^^,^^16^ The link between systemic inflammation and tumour behaviour implies the identification of clinically available surrogate markers to determine the extent of the disease.^17^^–^19

The Prognostic Index (PI) was first described in a cohort of patients with advanced non-small-cell lung cancer, where it showed a significant value for survival prognosis. The PI can easily be calculated from CRP serum concentration and white blood count (WBC) derived preoperatively.^20^ Yet, its impact on survival in a selected cohort of patients with PDAC undergoing resection has not been investigated to date.

The purpose of the present study was to assess the utility of the PI as a prognostic biomarker for cancer-specific survival in patients with PDAC undergoing resection with due regard to well-established prognostic factors, such as CA19-9 and tumour stage. We further intended to assess whether factors that interact with the immune and inflammation cascade might interfere with the PI’s strength for survival prognostication.^21^^–^31

## Methods

### Study Cohort

The study cohort consisted of patients with PDAC who underwent pancreatic resection at a tertiary referral University hospital. Patients were prospectively entered into an institutional database and followed over time. Data analysis was retrospectively performed, including only those patients who successfully underwent pancreatic resection. Patients with borderline resectable disease who received neoadjuvant chemo- or (radio-)chemotherapy of various protocols also were included.^5^ Hereby, all patients completed the full course of neoadjuvant therapy. Patient follow-up was completed by August 2017. Histopathological tumour characteristics were determined according to the AJCC/UICC TNM staging system of resected surgical specimen.^32^ Patients with any kind of obvious infection 6 weeks before surgery as well as patients with distant metastases (AJCC/UICC stage IV) were excluded from the study. Examinations were conducted in accordance with the Helsinki declaration. Approval was obtained from the local Ethics Committee (EK-Nr. 1166/2013).

### Calculation of the Prognostic Index

The Prognostic Index (PI) combines C-reactive protein (CRP) and white blood cell count (WBC), which were routinely measured during preoperative evaluation 2 days before resection, when patients were admitted for surgery. Following the previous description, patients were divided into three PI groups as summarized in Table 1.^20^ CRP serum concentration was measured automatically by Cobas^®^ 8000 modular analyser series (Roche^®^), with normal levels below 10 mg/L. WBC was analysed by Sysmex^®^ XE-5000 (Sysmex^®^), normal range 4.0 to 10.0 G/L.

**Table 1:** Definition of the Prognostic Index consisting of preoperative C-reactive protein and white blood count

Prognostic Index	*n* (%)	CRP (mg/L)	WBC (G/l)
0	235 (65.8)	≤ 10	≤ 11
1	108 (30.3)	≤ 10	> 11
		> 10	≤ 11
2	14 (3.9)	> 10	> 11

### Statistical Analysis

Numerical data were described by median (range) and categorical variables by frequencies. The Wilcoxon rank-sum test was applied to compare patient group PI, WBC, and CRP values. The Spearman rank-correlation coefficient (*r*_s_) was calculated for the correlation between the PI and ordinal or continuous variables. The inverse Kaplan–Meier method was used to estimate the median follow-up time.^33^ Survival estimates were calculated using the Kaplan–Meier method including the log-rank test for group comparisons. Surgical mortality was defined as death within the median length of hospital stay (12 ± 4 days); within this period only two patients died (day 6 and day 11, respectively). Cancer-specific survival was defined as the time period from pancreatic surgery to death from PDAC. In order to identify independent predictive factors for survival, established prognostic factors and factors that are known to influence immune- and inflammation status were entered into univariate and multivariable Cox regression models in addition to the PI.^2^^,^^5^^,^^21^^–^26^,^^32^ To examine explicitly the factors that may interfere with the PI’s strength for survival prognostication, an interaction model was built, considering interaction terms of the potential prognostic factors with the PI, for inclusion, by using the forward stepwise method. For all analyses, a two-sided *p-*value < 0.05 was considered statistically significant. Statistical analysis was performed in IBM SPSS Statistics Version 24 software (IBM©, USA) for macOs Sierra (Apple Inc.©, USA) and SAS Version 9.4 (SAS Institute Inc. (2016), Cary, NC).

## Results

### Patient and Tumour Characteristics in Relation to the PI

Medical records of 357 patients who underwent pancreatic resection for PDAC were analysed. Patient and tumour characteristics are shown in Table 2. Of the 357 patients comprising the study cohort, 235 (72.1%) patients underwent pylorus-preserving pancreaticoduodenectomy, and in 72 (23.5%) patients, distal pancreatectomy was performed. Tumour stage was distributed as follows: 13 patients had stage IA, 25 patients stage IB, 45 patients stage IIA, 255 patients stage IIB, and 19 patients stage III disease according to UICC/AJCC TNM staging system.^32^ Hence, 274 of 357 (76.8%) had a nodal-positive disease. Patients with borderline or locally advanced PDAC were referred to neoadjuvant therapy; depending on the patients’ condition, either bevacizumab, 5-fluorouracil-, or gemcitabine-based protocols were given. Of the 50 patients who received neoadjuvant therapy, 41 patients (82.0%) received gemcitabine-based therapy, and 9 (18.0%) patients received 5-fluorouracil-based therapy. During a median follow-up time of 100.5 (range 0–198) months, 333 (93.3%) patients died from their disease or disease-related complications; only 2 patients died within a median length of hospital stay of 12 days (± 4 days). The median cancer-specific survival time was 16.5 (range 0.2–198) months. PI score groups were distributed as follows: PI 0 if CRP ≤ 10 mg/L and WBC ≤ 11 × 10^9^; PI 1 if one of the two markers was elevated; and PI 2 if both markers were elevated (Table 1). The relation of the PI, with patient and tumour characteristics, is shown in Table 2. In addition, WBC was significantly higher in patients with a positive smoking history (*p *= 0.004), but not in patients with a history of diabetes (*p *= 0.36), and did not correlate with tumour stage (UICC, *r*_s_ = 0.07, *p *= 0.19). Patients with nodal-positive disease did not differ with respect to WBC compared with nodal-negative patients (*p *= 0.42; data not shown). Furthermore, CRP did not correlate with the patients’ smoking history (*p *= 0.90). With respect to neoadjuvant therapy, all patients with a PI of 2 (*n* = 2, 100.0%) received a gemcitabine-based regimen, while patients with a PI of 1 and 0 received 5-fluorouracil- or gemcitabine-based regimen (PI 1: *n* = 1, 14.3% or *n* = 6, 85.7%; PI 0: *n* = 8, 19.5% or *n* = 33, 80.5%). We found no significant difference in the PI between the two groups of neoadjuvant therapy regimens given (*p *= 0.55).

**Table 2:** Patient and tumour characteristics in relation to the Prognostic Index

Factors		PI group 0	PI group 1	PI group 2	*p-*value
Study cohort	357 (100)	235 (65.8)	108 (30.3)	14 (3.9)	
Sex					0.17*
Male, *n* (%)	186 (52.1)	117 (49.8)	59 (54.6)	10 (71.4)	
Female, *n* (%)	171 (47.9)	118 (50.2)	49 (45.4)	4 (28.6)	
Age					*r*_s_ = 0.06** (0.25)
Median (range)	76 (37–101)	76 (47–101)	77 (37–97)	74 (62–93)	
Diabetes					0.74*
Yes, *n* (%)	80 (22.4)	55 (23.4)	19 (17.6)	6 (42.9)	
No, *n* (%)	277 (77.6)	180 (76.6)	89 (82.4)	8 (57.1)	
Smoking history					0.17*
Yes, *n* (%)	153 (42.9)	94 (40.0)	54 (50.0)	5 (35.7)	
No, *n* (%)	204 (57.1)	141 (60.0)	54 (50.0)	9 (64.3)	
Bile stent					0.56*
Yes, *n* (%)	172 (48.2)	115 (48.9)	53 (49.1)	4 (28.6)	
No, *n* (%)	185 (51.8)	120 (51.1)	55 (50.9)	10 (71.4)	
Neoadjuvant therapy					0.01*
Yes, *n* (%)	50 (14.0)	41 (17.5)	7 (6.5)	2 (14.3)	
No, *n* (%)	307 (86.0)	194 (82.5)	101 (93.5)	12 (85.7)	
Neoadjuvant tx protocol					0.55*
Gemcitabine-based tx	41 (82.0)	33 (80.5)	6 (85.7)	2 (100.0)	
5-fluorouracil-based tx	9 (18.0)	8 (19.5)	1 (14.3)	0 (0.0)	
Preoperative bilirubin					*r*_s_ = 0.22** (*p *< **0.0001**)
Median (range)	1.10 (0–68)	0.81 (0–68)	2.02 (0–48.9)	0.93 (0.2–17.9)	
Preoperative CA 19-9					*r*_s_ = 0.07** (*p* = 0.19)
Median (range)	133 (0–10,280)	125.1 (0–10,280)	141.7 (0–2837)	319.9 (4.6–2995)	
Type of surgery					0.69*
PPPD	235 (72.1)	129 (42.0)	98 (31.9)	8 (2.6)	
DP	72 (23.5)	37 (12.1)	31 (10.1)	4 (1.3)	
Tumour grading					*r*_s_ = − 0.002** (*p* = 0.97)
Good (G1), *n* (%)	15 (4.2)	10 (4.3)	4 (3.7)	1 (7.1)	
Moderate (G2), *n* (%)	225 (63.0)	148 (63.0)	68 (63.0)	9 (64.3)	
Poor (G3), *n* (%)	117 (32.8)	77 (32.7)	36 (33.3)	4 (28.6)	
AJCC/UICC stage					*r*_s_ = 0.12** (*p* = **0.03**)
< IIB, *n* (%)	83 (23.2)	62 (26.4)	18 (16.7)	3 (21.4)	
≥ IIB, *n* (%)	274 (76.8)	173 (75.6)	90 (83.3)	11 (78.6)	
Resection margin					*r*_s_ = 0.009** (*p* = 0.86)
Negative (R0), *n* (%)	286 (80.1)	188 (80.0)	89 (82.4)	9 (64.3)	
Positive (R1 + 2), *n* (%)	71 (19.9)	47 (20.0)	19 (17.6)	5 (35.7)	

Bold values are statistically significant (*p*-value < 0.05)

*Tx* therapy; *PPPDP* pylorus-preserving pancreaticoduodenectomy; *DP* distal pancreatectomy

*Wilcoxon rank-sum test (*p-*value)

**Spearman correlation coefficient (*r*_s_, *p-*value)

### Survival Analysis, Prognosis Estimation, and Interaction Model

**Fig. 1:**
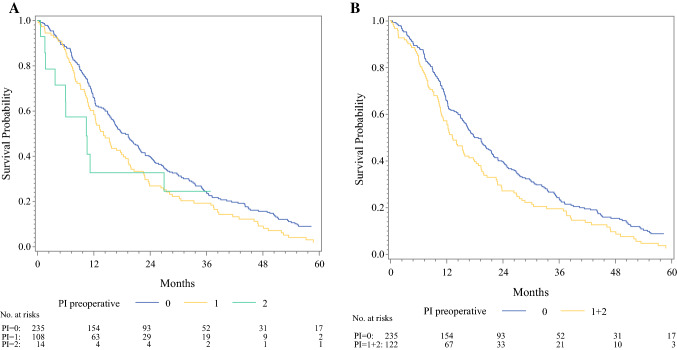
**a** Kaplan–Meier survival curves for PI groups: high preoperative PI score impairs survival in patients with resectable PDAC (PI 0 vs. PI 1. vs. PI 2, *p *= 0.044). **b** Kaplan–Meier survival curves for PI groups: high preoperative PI score impairs survival in patients with resectable PDAC (PI 0 vs. PI 1 + 2, *p *= 0.012)

Comparison of the PI groups using Kaplan–Meier curves showed significantly different survival times (*p *= 0.044; Fig. 1a). When comparing pooled analysis between patients without elevated inflammatory factors (PI 0) and patients with elevated inflammatory factors (PI 1 and 2), the differences in survival were higher (*p *= 0.012, Fig. 1b). Median (quartiles) patient survival with a high PI (group 1 + 2) was 13.2 months (7.7–27.0) compared with 18.7 months (10.2–35.4) in patients with a low PI (group 0). Univariate Cox-Regression analysis confirmed that the PI had a significant impact on survival (*p *= 0.013) where the risk for cancer-specific death was 1.33 times higher (CI 1.06–1.67; data not shown) in the group with a high PI (PI 1 + 2 vs. PI 0). When including the PI into a multivariable Cox-Regression model in addition to established prognostic factors and factors that interfered with the immune and inflammation cascade, the PI proved to be a strong and independent prognostic factor for survival (*p *= 0.003) next to tumour grading (*p *= 0.009), age (*p *= 0.019) and neoadjuvant therapy (*p *= 0.0009; Table 3). Furthermore, the interaction terms were included in the multivariable model; what became evident was that the presence of a bile stent had an influence on the strength of the PIs survival prognosis (*p *= 0.032; Table 4). For patients without a bile stent, the hazard ratio was estimated as 1.13 (CI 0.81–1.57), whereas a hazard ratio of 1.87 (CI 1.34–2.61) was estimated for patients with a bile stent (data not shown). To test whether advanced disease has to be treated as a separate entity with respect to the PI, we performed a sensitivity analysis in a cohort of 307 patients with primary resectable PDAC only; we found that the PI had a significant impact on survival (*p *= 0.007); the risk of cancer-specific death was 1.40 times higher (CI 1.10–1.78; data not shown) in the group with a high PI (PI 1 + 2 vs. PI 0). Included in the multivariable Cox-Regression model, the PI persisted as a strong independent prognostic factor for postoperative survival (*p *= 0.007) next to tumour grading (*p *= 0.016) and stage (*p *= 0.009; Table 5). When including the interaction terms into the multivariable model, again the presence of a bile stent had an influence on the strength of the PIs survival prognosis (*p *= 0.018) in contrast to the other concomitant factors (Table 6).

**Table 3:** Cox-Regression analysis in PDAC patients undergoing resection (*n* = 357)

Pre-/postoperative factor	Univariate analysis	Multivariable model		
	*p*-value	*p*-value	Hazard ratio	95% Confidence interval
Prognostic Index (1 + 2 vs. 0)	**0.013**	**0.003**	1.44	1.13–1.83
Tumour grading	**0.019**	**0.009**	1.32	1.07–1.62
AJCC/UICC stage	**0.053**	0.124	1.12	0.97–1.29
Age	0.079	**0.019**	1.01	1.00–1.02
Diabetes	0.178	0.086	1.26	0.97–1.65
Smoking history	0.514	0.408	1.10	0.88–1.37
Bile stent	0.717	0.528	1.07	0.86–1.34
Neoadjuvant tx	0.046	**0.0009**	1.73	1.25–2.40
Preoperative bilirubin (log2-transformed)	0.654	0.582	0.99	0.94–1.04
Preoperative CA 19-9 (log2-transformed)	0.138	0.070	1.03	1.00–1.07
Resection margin	0.048	0.029	1.36	1.03–1.79

Bold values are statistically significant (*p*-value < 0.05)

**Table 4:** Interaction terms considered for inclusion in the multivariable Cox-Regression model

Pre-/postoperative factor	*p*-value
PI * Tumour grading	0.871
PI * AJCC/UICC stage	0.334
PI * Age	0.227
PI * Diabetes	0.066
PI * Smoking history	0.563
PI * Bile stent	**0.032**
PI * Neoadjuvant tx	0.663
PI * Preoperative bilirubin	0.740
PI * Preoperative CA 19-9	0.225
PI * Resection margin	0.351

Bold values are statistically significant (*p*-value < 0.05)

**Table 5:** Cox-Regression analysis in patients with primary resectable PDAC (borderline resectable/locally advanced PDAC excluded; *n* = 307)

Pre-/postoperative factor	Univariate analysis	Multivariable model		
	*p*-value	*p*-value	Hazard ratio	95% Confidence interval
Prognostic Index (1 + 2 vs. 0)	**0.007**	**0.007**	1.43	1.11–1.85
Tumour grading	**0.011**	**0.016**	1.33	1.06–1.68
AJCC/UICC stage	**0.003**	**0.009**	1.26	0.06–1.51
Age	0.080	0.023	1.01	1.00–1.02
Diabetes	0.105	0.051	1.33	1.00–1.77
Smoking history	0.690	0.450	1.10	0.87–1.40
Bile stent	0.766	0.482	1.09	0.86–1.39
Preoperative bilirubin (log2-transformed)	0.252	0.910	1.00	0.94–1.06
Preoperative CA 19-9 (log2-transformed)	0.174	0.143	1.03	1.00–1.07
Resection margin	0.056	0.073	1.30	0.98–1.74

Bold values are statistically significant (*p*-value < 0.05)

**Table 6:** Interaction terms considered for inclusion in the multivariable Cox-Regression model

Pre-/postoperative factor	*p*-value
PI * Tumour grading	0.829
PI * AJCC/UICC stage	0.138
PI * Age	0.265
PI * Diabetes	0.074
PI * Smoking history	0.497
PI * Bile stent	**0.018**
PI * Preoperative bilirubin	0.702
PI * Preoperative CA 19-9	0.212
PI * Resection margin	0.426

Bold values are statistically significant (*p*-value < 0.05)

## Discussion

The present study assessed the preoperative conducted PI applicability for survival prognostication in patients with PDAC undergoing resection. The PI was found to be a strong independent prognostic factor for survival that was superior to other prognostic factors, such as tumour grade, stage, and CA 19-9. In this analysis, of all concomitant factors that interact with the immune and inflammation cascade, the presence of a bile stent was the only factor that influenced the PI’s strength for survival prognostication.^21^^–^31

Prognosis estimation in PDAC patients undergoing resection mainly relies on postoperative available factors. The AJCC/UICC tumour stage, which becomes available only after histopathological reworking, provides the basis for subsequent treatment decisions.^2^^,^^32^ To date, postoperative survival is not routinely estimated before treatment of PDAC. CA 19-9 is the only biomarker available for prognostication, yet the high specificity limits its utility and definitive applicability with respect to treatment response can only be determined during treatment course in terms of marker decrease.^34^ In addition, we show that the PI proves highly significant as an independent prognostic factor in contrast to CA 19-9. Consequently, the investigation of screening biomarkers that can be reliantly applied before surgery is of the utmost importance.

Cancer-related inflammation is a hallmark of cancer.^35^ Next to tumour proliferation, maintenance and dissemination, it promotes angiogenesis, disarrangement of adaptive immunity as well as impaired chemotherapy response.^36^ Accordingly, specific scores have been established on the basis of inflammatory markers that have been validated as prognosticators of therapy response and outcome in patients with solid malignancies.^6^^,^^7^^,^^9^^,^^17^^,^^18^^,^^20^^,^^37^^–^41 However, some of these prognostic scores require extended analysis of differential blood cell count, which is not routinely measured in patients with primary PDAC undergoing resection.^6^^–^8^,^^10^^,^^18^^,^^40^^,^^41^ The PI involves CRP and WBC as prognostic biomarkers. CRP has demonstrated superior prognostic impact in survival estimation of cancer patients compared with WBC components and albumin.^40^^,^^42^ High CRP levels reflect the risk of cancer incidence and cancer-associated death.^42^^,^^43^ The PI and the (modified) Glasgow Prognostic Score, made up of CRP and albumin, are powerful prognostic tools in a variety of cancers compared with the neutrophil–lymphocyte ratio (NLR), the platelet-lymphocyte ratio (PLR), and the prognostic nutritional index (PNI).^40^^,^^41^ With regards to PDAC, neutrophilia in particular has been associated with pro-cancer effects.^44^ In the present analysis, a strong correlation between WBC and tumour stage was found, with higher WBC counts in patients with nodal-positive disease. These findings reflect the utility of leucocytes as valid biomarkers for patients with PDAC undergoing resection. Consequently, the combination of both markers, CRP and WBC, might most reliably reflect the inflammatory status of the patients allocated to surgery. Another advantage of the PI is its cost effectiveness and availability during initial routine blood tests.^20^ In contrast, scores, such as the neutrophil–lymphocyte ratio (NLR), the platelet-lymphocyte ratio (PLR), and the prognostic nutritional index (PNI), use parameters that need to be obtained by differential blood tests.

Previous analyses of PDAC specimens after neoadjuvant treatment have yielded conflicting results regarding the state of cancer-related inflammation. However, neoadjuvant treatment seems to induce an altered immune response that influences survival.^28^^,^^30^^,^^31^^,^^45^ We show that the PI’s prognostic strength is not impaired by neoadjuvant therapy. Of note, one might speculate to exclude patients with borderline resectable or locally advanced PDAC from this analysis due to the different treatment setting; however, in this study, we wanted to test the influence of concomitant factors (and neoadjuvant treatment as such) on the PI’s strength on survival prognostication. Still, in a subgroup analysis with primary resectable patients only (*n* = 307), the PI persisted as solid and strong independent factor for survival. Next to chemotherapy, further concomitant factors such as age, diabetes, smoking and high bilirubin levels common in the PDAC patient population, have been shown to interact with the immune- and inflammation cascade and thus might influence the PI’s prognostic strength.^21^^–^31 We demonstrate that the only factor that influences the PI’s strength for survival prognostication is the presence of a bile stent. In contrast to risk factors that are chronically present (age, diabetes, smoking), preoperative introduction of a bile stent fosters (incipient) cholangitis. Although patients who had a bile stent did not suffer from higher PI levels (compared with those without bile stent), bacterial overgrowth caused by the intervention maintains (chronic) inflammation, which on the one hand might not impair CRP and WBC levels but on the other hand hampers physical reserves and thus postoperative survival.^46^^,^^47^ However, despite the presence of PDAC-specific risk factors, progressive tumour mass (CA 19-9) as well as tumour stage, the PI is a reliable tool for preoperative survival prognostication in PDAC patients undergoing resection.

To our knowledge, this is the first study to assess the PI’s clinical utility for survival estimation in a selected cohort of patients with PDAC undergoing resection. The results indicate that the PI is a reliable tool in predicting disease-specific survival prior to surgery. In addition, the PI may perform better than other scores for survival prognostication, most likely due to the combination of CRP and WBC and its robustness against other concomitant factors. Nonetheless, this study is of retrospective nature and reflects an unicentre experience. Thus, the above findings require validation by a prospective, multicentre protocol.

In conclusion, the determination of the PI as a prognostic biomarker for systemic inflammatory activity offers an opportunity to identify patients at risk for poor survival prior to surgery. However, prospective multicentre studies are required to prove this concept.
